# Dietary Composition Independent of Weight Loss in the Management of Non-Alcoholic Fatty Liver Disease

**DOI:** 10.3390/nu9080800

**Published:** 2017-07-26

**Authors:** Tannaz Eslamparast, Puneeta Tandon, Maitreyi Raman

**Affiliations:** 1 Division of Gastroenterology, Department of Medicine, University of Alberta, Edmonton, AB T6G 2X8, Canada; eslampar@ualberta.ca (T.E.); ptandon@ualberta.ca (P.T.); 2 Cirrhosis Care Clinic and CEGIIR, University of Alberta, Edmonton, AB T6G 2X8, Canada; 3 Division of Gastroenterology, Department of Medicine, University of Calgary, Calgary, AB T6G 2X8, Canada

**Keywords:** non-alcoholic fatty liver disease, dietary composition, Mediterranean diet

## Abstract

Poor dietary composition is an important factor in the progression of non-alcoholic fatty liver disease (NAFLD). The majority of NAFLD patients follow diets with overconsumption of simple carbohydrates, total and saturated fat, with reduced intake of dietary fiber and omega-3 rich foods. Although lifestyle modifications including weight loss and exercise remain the keystone of NAFLD management, modifying dietary composition with or without a calorie-restricted diet may also be a feasible and sustainable strategy for NAFLD treatment. In the present review article, we highlight the potential therapeutic role of a “high quality healthy diet” to improve hepatic steatosis and metabolic dysfunction in patients with NAFLD, independent of caloric restriction and weight loss. We provide a literature review evaluating the evidence behind dietary components including fiber-, meat- and omega-3-rich diets and, pending further evidence, we concur with the EASL-EASD-EASO Clinical Guidelines recommendation of the Mediterranean diet as the diet of choice in these patients.

## 1. Introduction

Non-alcoholic fatty liver disease (NAFLD) is one of the most common non-communicable diseases worldwide, affecting 25–30% of the global population, with the highest prevalence in the Middle East and South America. In North America and Europe, one-fourth of the adult population has NAFLD [1,2]. The major established risk factors for NAFLD include obesity, insulin resistance and dyslipidemia [3]. The NAFLD spectrum ranges from simple liver steatosis to hepatic necroinflammation called non-alcoholic steatohepatitis (NASH) with or without fibrosis. This may progress to advanced cirrhosis and hepatocellular carcinoma (HCC) [4,5]. NAFLD acts in synergy with related metabolic risk factors and other existing conditions to promote the development of HCC [6], and importantly, HCC can be seen in the absence of cirrhosis in this condition [7]. The burden of NAFLD continues to increase. It is the third leading cause of liver transplantation and, by 2030, it is predicted to become the most common reason for transplantation in the United States [8].

The majority of NAFLD patients overconsume fructose, red meat and saturated fat [9,10]. In addition they have reduced intake of dietary fiber and omega-3 rich food [9,10]. This pattern of dietary intake can lead to obesity and the metabolic syndrome, increasing the risk for NAFLD. In addition, there is emerging evidence that a deleterious dietary composition may predispose people to NAFLD, even in the absence of obesity [11,12].

Lifestyle modifications involving weight loss and exercise remain the cornerstone of NAFLD management [13]. Caloric restriction is the most important element in nutrition intervention for NAFLD patients and plays a critical role in visceral, subcutaneous and hepatic fat reduction, and weight loss [14]. Weight reduction of 3–5% can cause a notable decrease in hepatic fat and improvement in insulin sensitivity [15]. However, weight loss and weight maintenance remain a considerable challenge for many individuals. Conceivably, modifying dietary composition with or without a calorie-restricted diet may be a feasible and sustainable strategy for NAFLD management. As such, the present review focuses on addressing the potential role for modifying dietary composition and dietary patterns in the management of NAFLD—interventions that may have efficacy with or without weight loss.

## 2. Definition of NAFLD

NAFLD is a hepatic manifestation of the metabolic syndrome [16]. It encompasses a broad pathological spectrum of disease characterized by excessive hepatic triacylglycerol (TAG) deposits in more than 5% of hepatocytes in patients with little or no history of alcohol consumption [4,13].

## 3. Pathophysiology of NAFLD

The pathophysiology of NAFLD is complex and includes numerous genetic, dietary, metabolic and hormonal factors. In 1998, Day and James proposed the “2-hit hypothesis” [17] to explain the progression from steatosis to NASH. New insights suggest a multiple hit hypothesis, with the first hit of insulin resistance leading to increased fat accumulation within the hepatocyte. The steatotic liver is vulnerable to lipid peroxidation, impaired hepatocyte apoptosis and increased cytokine activity [17]. In addition, a complex interplay between multiple factors such as oxidative stress-mediated lipotoxicity, proinflammatory adipokines and mitochondrial dysfunction act in parallel with genetic predisposition to advance disease. Dietary carbohydrate (CHO) intake and insulin resistance may inhibit the reduction of reactive oxygen species and represent potential additional hits in a susceptible individual [18]. This multiple hit theory provides a broader pathophysiologic view of NAFLD compared to the “two-hit” theory [19,20].

### 3.1. Lipid Metabolism

Obesity and insulin resistance can result in an elevated free fatty acid (FFA) flux induced by dysregulation of de novo lipogenesis (DNL), lipolysis of visceral fat, and dietary fat. Increased hepatic FFA flux leads to excess fat deposition in hepatocytes with a large metabolic load and promote hepatocyte lipotoxicity and oxidative stress [21]. Ultimately, the additive effects of cytokines released by Kupffer cells and activation of the immune response and cell death pathways meditate the transition to NASH [22,23].

### 3.2. Glucose Metabolism

CHO intake can also stimulate hepatic lipogenesis by converting excess glucose to fatty acids. Under normal conditions, insulin transports glucose into the cell to be stored as glycogen [24]. Hepatic steatosis occurs when impaired insulin signaling results in compensatory hyperinsulinemia. In this situation, glucose absorption decreases in skeletal muscle providing further substrate for DNL. Hyperinsulinemia also decreases glycogen synthesis, increases hepatic FFAs uptake, alters TAG transportation, and inhibits β-oxidation [22,25].

### 3.3. Gut Microbiome

The gut microbiota has been implicated in obesity, diabetes, metabolic syndrome and NAFLD through effects on calorie salvage, host energy metabolism, proinflammatory signaling, and via direct hepatotoxicity of bacterial products [26]. Obese humans may have a fundamentally different gut microbiota, with decreased diversity, but increase in the representation of the phylum Firmutes and proportionate decrease in Bacteroidetes compared with lean individuals [27]. Microbes produce several hundred volatile organic compounds, including ethanol, of which the systemic effects are unknown [28]. Our group has previously shown that in obese patients with clinically suspected NAFLD, a significantly altered fecal volatile organic compound profile and compositional shift in the fecal microbiome is observed [29]. In this study, we were unable to replicate previous observations of phylum-level differences in the fecal microbiota. Additional study findings revealed that ester volatile organic compounds were identified more frequently in NAFLD compared to controls. Conceivable the volatile organic compounds are of bacterial origin, however, the role of diet in the genesis of volatile organic compounds cannot be excluded [30]. The gut-liver axis has a pivotal role in the development and progression of NAFLD [31]. Gut-derived lipopolysaccharides (LPS), a component of gram-negative bacteria induces liver injury by invoking a cascade of morphological, biochemical and functional changes such as insulin resistance, increased hepatic oxidative stress, acute inflammatory responses, metabolic deregulation and fibrosis [32,33]. Bacterial translocation is a process in which alterations in tight junction proteins reduce the intestinal barrier integrity, resulting in increased endotoxin absorption. In a healthy individual, these bacterial products are rapidly cleared by the hepatic immune system, but in an injured liver, they can result in the release of reactive oxygen metabolites, proteases and other degenerative enzymes, which worsen liver damage [34,35]. Over the last decade, increased gut permeability and elevated levels of LPS in the serum have been documented in NAFLD and NASH patients [36]. Gut dysbiosis therefore plays a role in the development and progression of liver damage and in the severity of NAFLD, NASH and their complications [37].

### 3.4. Visceral Adipose Tissue vs. Subcutaneous Adipose Tissue (VAT: SAT)

Adipose tissue is a complex, active endocrine organ. It contributes to the pathogenesis of NAFLD by modulating insulin sensitivity by secreting adipocytokines. Visceral fat tissue (VAT) is densely infiltrated with macrophages, promotes inflammation, stimulates proinflammatory cytokine secretion such as TNF-α, and Interlukin-6 (IL-6), and inhibits insulin sensitivity of adipocytes [22]. Expression of TNF-α is greater in VAT compared to subcutaneous fat (SAT), and can trigger proinflammatory pathways [38], which disrupt insulin signaling and increase steatosis. Conversely, adiponectin, a protein hormone with anti-inflammatory properties, produced by adipocytes, has decreased secretion in VAT, leading to altered lipid metabolism and increased inflammation [22].

## 4. Observations of Dietary Composition and Patterns in NAFLD

### 4.1. Calorie, Macronutrient Composition and Dietary Fiber

The relationship between diet and the development of NAFLD is complex and extends beyond total energy intake. Assessment of total energy intake in patients with NAFLD compared with controls demonstrates discordant results. Although some investigators have shown higher energy intake in NAFLD compared to healthy controls [39], others have identified similar energy intake in both groups, with notable differences in dietary composition [40]. Cortez-Pinto and colleagues identified significantly lower protein and CHO, and higher total fat and *n-*6 consumption in NAFLD patients compared to controls [40]. Musso et al. showed that patients with NASH compared to controls had non-significantly higher intake of total fat; in contrast, higher intake of saturated fat, and lower PUFA consumption was observed in NASH compared to controls [11]. Recently, Wehmeyer and colleagues found no differences in the consumption of fat between NAFLD patients and controls [39]. The divergent study results may arise due to heterogeneous patient populations (NAFLD vs. NASH) and/or varying dietary data capture tools.

Several studies have focused on the detrimental effects of high consumption of simple CHO on hepatic and metabolic functions in NAFLD [9,15]. Recently, a study observed a significantly higher glucose intake per 1000 kcal in patients with NAFLD compared to controls [39]. In 2007, a group of investigators reported that simple CHO consumption, particularly from soft drinks, in subjects diagnosed with NAFLD was significantly greater than in subjects with a normal liver (23 ± 42 vs. 12 ± 24 g/day, *p*-value = 0.03). The result was sustained even after adjusting for sex, age, BMI and total calorie intake (OR = 1.45, 95% CI 1.13–1.58) [10]. Volynets identified a higher intake of protein, total carbohydrates, monosaccharides and disaccharides compared with controls [41]. A study of 28 patients with NASH and 18 patients with simple steatosis supported that a diet rich in CHO, particularly sweets and not cereals, was associated with higher risk of NASH compared to simple steatosis [42]. Although, this study found a significant association in both sexes, another study in 2015 reported that a high-CHO/sweet dietary pattern, characterized by high intakes of fruit, cakes, soft drinks and sugary snacks, was positively associated with risk of NAFLD only in females. They also showed that females in the highest quartile had a greater risk of developing NAFLD than those in lowest quartile, after adjustment for cofounding factors (OR = 2.19, 95% CI 1.4–3.46), while no association was observed in males [43]. These findings are consistent with the observation in the study by Tajitma et al. [44], who investigated the relationship between CHO intake and NAFLD prevalence in middle-aged Japanese.

Regarding the protein content of the diet, several studies have reported a significantly higher protein intake in patients with NAFLD compared to controls [10,39,40,41], while other studies found no difference [11,45]. In 2016, Kobayashi, et al. [46] compared the dietary intake of Japanese patients with NAFLD and T2D. Their results showed a significantly higher energy ratio from protein in NAFLD patients than in T2D (*p* = 0.008).

Poor fiber intake is common in NAFLD [39,40]. In a series of 25 NASH patients and 25 matched healthy controls, the mean daily fiber intake was almost 50% lower in NASH versus control patients (13 ± 4 g vs. 23 ± 8 g, *p* = 0.0001) [11]. This is supported by a subsequent study showing lower fiber intake in NAFLD patients as compared to controls [39]. In a study from Japan, patients with NAFLD had a lower vegetable intake and a higher consumption of fruits and sweets compared to T2D patients [46]. In 2016, Cheng and colleagues [45] assessed the relationship between dietary intake and hepatic lipid content in NAFLD, and showed that fiber intake was inversely associated with hepatic fat fraction and intrahepatic lipid.

Taken together, the macronutrient composition of the diet, particularly high independent consumption of CHO, simple sugars, fat and protein, and low fiber intake may be associated with the risk of NAFLD, independent of energy overconsumption. The mechanism by which the intake of certain nutrients may influence the disease has not yet been completely understood.

### 4.2. Meat (Red Meat vs. Other)

High meat intake is associated with glucose intolerance, impaired insulin sensitivity, and may increase the risk of T2D [37,47]. Although T2D is accepted as a risk factor for NAFLD, there are no data investigating the direct association between intake of dietary protein sources and NAFLD. A recent cross-sectional study [10] showed that subjects with NAFLD consumed 27% more protein from all types of high and low fat meat (beef, liver, sausage, lamb, chicken and turkey) compared to subjects without NAFLD. Even after adjusting for sex-, age-, BMI- and total calorie intake-, a higher protein intake resulted in a higher risk of NAFLD. Interestingly, when the type of protein intake was considered, the intake of protein from fish high in omega-3 trended to decrease the risk of NAFLD (OR = 0.73, 95% CI 0.52–1.04). Although, this study found no significant difference in the consumption of different types of meat and fats, another case-control study reported that the intake of red meat was significantly higher in NAFLD cases compared to controls [48]. Notably, in the latter study, the meat consumption in both study groups exceeded the recommendation level of Chinese Dietary Pagoda (75 g/day). Findings showed no significant differences in white meat consumption between NAFLD and control groups [48].

High intake of processed meat has also been associated with an increased risk of NAFLD. This may be linked to the high content of sodium, saturated and trans-fatty acids in these products [49,50].

There are several possible explanations for the potential role of red meat intake in NAFLD: (a) red meat is high in saturated fat and cholesterol; (b) presence of preservatives and additives in processed meat; (c) presence of other dietary factors with negative effects in individual with high meat intake, since they usually follow a “Western” dietary pattern, which has been linked to hypertension, diabetes, obesity and certain cancers [47,51,52].

### 4.3. Omega-3 Rich Diets

Dietary sources of omega-3 PUFAs, especially eicosapentaenoic acid (EPA) and docosahexaenoic acid (DHA) are found in with highest concentration in seafood, mostly fatty fishes including pilchards, sardines, mackerel, trout, salmon, herring and tuna. Marine omega-3 PUFAs are also found in lean fish (haddock, cod, and crustaceans), shellfish, and fish oils with lower concentrations [53]. Omega-3 PUFAs are also found in certain vegetable oils such as flaxseed oil. Studies have associated a higher consumption of fish rich in omega-3 with a reduced risk of NAFLD [10]. This has been supported by case-control studies reporting a significantly lower concentration of omega-3 PUFA, especially DHA, in NAFLD patients than in healthy individuals [54,55,56]. There are few data concerning hepatic fatty acid composition in patients with NAFLD. In one study, NAFLD patients had a depletion of omega-3 PUFAs in hepatic TAG. The low level of circulating omega-3 PUFAs was associated with greater rates of lipogenesis and hepatic FFA flux along with a reduction in VLDL synthesis and β-oxidation, promoters of NAFLD to its necroinflammatory form, NASH [54]. Similarly, a study by Allard et al. [30] compared three groups of patients (NASH, elevated liver enzymes with minimal histological findings and patients with simple steatosis). They showed that NASH patients had a total PUFA intake below the recommended level and lower hepatic omega-3 and omega-6 compared to the other two groups. There were no differences in *n*-6/*n*-3 ratio among the three study groups [30].

## 5. Dietary Intervention on NAFLD Outcomes

Lifestyle modification is the first-line treatment option, with weight loss via a hypocaloric diet being the most important therapeutic target in NAFLD [57]. There is evidence in support of particular diets, most notably the Mediterranean diet (Table A1) [58].

### 5.1. Mediterranean Diet

A “Mediterranean diet” (MD) is a dietary pattern originally inspired by the traditional diet in Mediterranean countries, characterized by a high consumption of plant-based foods, such as vegetables, fruits, whole grains, seeds, nuts and legumes, and moderate consumption of protein-source foods such as fish and poultry (Table 1). This diet is rich in monounsaturated fatty acids (MUFA) primarily from olive oil and olives, reduced fat dairy products and low red meat intake. It has proven beneficial effects on the prevention of cardiovascular disease, hypertension, hypercholesterolemia, and obesity [59,60]. In 2014, a small observational study explored the potential association between a Mediterranean diet and the presence and the severity of NAFLD. Kontogianni et al. [61] found that a greater adherence to the Mediterranean diet was significantly correlated with a lower degree of hepatic steatosis and insulin resistance in patients with biopsy-proven NASH compared to simple fatty liver. The MD score was negatively correlated with the serum alanine aminotransferase (ALT), insulin resistance index, and the severity of steatosis [61].

The Mediterranean diet has been shown to reduce hepatic fat and improve hepatic insulin sensitivity even without weight loss in NAFLD patients [62]. Adherence to Mediterranean diet has also been linked to less advanced NAFLD and a lower risk of having the metabolic syndrome in this patient population [58]. Two randomized trials have contributed the most to the evidence for a potential beneficial effect for the MD in NAFLD. In a randomized parallel-group design, 37 men and 8 women with T2D were assigned to a 28-week isocaloric diet (high CHO/high fiber or high MUFA). There was a greater decrease in hepatic steatosis in the MUFA compared to the high CHO diet (29% vs. 4%) [63]. Additionally, another randomized cross-over trial assessed the impact of a MD compared to a standard low-fat high-CHO in non-diabetic NAFLD patients. A significant reduction in hepatic steatosis was observed with the MD [64]. Accordingly, the MD has been recommended as the diet of choice for the treatment of NAFLD by the EASL-EASD-EASO Clinical Practice Guidelines [13,65]. There are multiple potential mechanisms for the observed benefits of the MD on the course of NAFLD. Extra-virgin olive oil (EVOO) is a frequently identified dietary component of the MD, containing over 30 phenolic compounds. Phenolic compounds are known to exhibit antioxidant properties [66]. In addition, EVOO has been shown to reduce postprandial blood glucose levels and LDL potentially mediated through glucagon like peptide 1 upregulation [65]. Through various mechanisms, *n-*3 fatty acids in fish oil have been shown to reduce hepatic lipid accumulation, improve insulin sensitivity and have anti-inflammatory effects [65]. Higher intake of fruits and vegetables can reduce the energy density of the diet contributing to an important role in weigh management. Moreover, polyphenols and carotenoids contained in fruits and vegetables display beneficial effects on metabolic homeostasis and exert anti-inflammatory and anti-fibrotic effects, in NAFLD models, while suppressing activation of hepatic stellate cells [67]. High-fiber diets and whole grains have the potential to beneficially influence gut microbiota composition, which can be of relevance to the gut liver axis in the progression of NAFLD [68].

It should also be noted that a Mediterranean diet may be associated with a Mediterranean lifestyle, which may include adequate rest periods, differences in sun exposure and optimal physical activity, all of which may have a positive but independent impact on the course of NAFLD. Future well-designed studies exploring the longer term impact of the MD on the spectrum of NAFLD are required.

### 5.2. DASH Diet

A “Dietary Approach to Stop Hypertension” (DASH) diet is a dietary pattern rich in vegetables, fruits, whole grains, low-fat dairy products, fish, poultry, nuts and legumes but with emphasis on low intake of sodium, total fat, saturated fat, cholesterol and added sugars (Table 1). The DASH diet is high in fiber, potassium, calcium and magnesium [69]. Although this dietary pattern was primarily designed for lowering blood pressure [69], by directly playing a beneficial role in insulin resistance and dyslipidemia [70] it has recently shown beneficial effects on other metabolic disorders including T2D [71], cardiovascular disease [72], and NAFLD [73,74]. A recent case-control study in 102 patients with newly diagnosed NAFLD and 204 matched controls evaluated the association between adherence to the DASH diet and the risk of NAFLD using a validated food frequency questionnaire. Although there was an inverse association between the DASH-style diet and risk of NAFLD, after adjusting for BMI and dyslipidemia the significance of this relationship disappeared [73]. In a recent eight-week RCT conducted in overweight and obese patients with NAFLD, the DASH diet resulted in a significant reduction in body weight, serum TG level, VLDL-cholesterol and liver enzymes, and concurrent improvement in insulin sensitivity, biomarkers of oxidative stress and inflammation (high sensitivity-CRP, nitric oxide (NO), glutathione (GSH) malondialdehyde) in the intervention group compared to controls [74].

Possible mechanisms for the protective role of the DASH diet include: (i) High fruit and vegetable consumption, resulting in higher intake of antioxidants [75], which may suppress the inflammatory processes in NAFLD; (ii) Higher intake of whole grains, which are associated with a lower risk of comorbidities linked to NAFLD [76]; (iii) Increased intake of nuts, which are rich in MUFAs, PUFAs and vitamin E, which may improve the serum lipid profile, reduce blood pressure, and have a favorable impact on oxidative stress [77]; (iv) Lower dietary intake of simple sugar. High sugar intake is associated with dyslipidemia, insulin resistance and central obesity [78]; (v) Higher intake of calcium and magnesium content of the DASH diet may have beneficial effects on insulin sensitivity and the anti-inflammatory response by decreasing oxidative activity and restoring anti-oxidative enzymes [79,80,81]. More studies are required in this field to determine the features of the DASH diet that are associated with the most benefit in patients with NAFLD.

### 5.3. Fiber Intervention (Soluble, Prebiotic)

Prebiotics are known as selectively fermented ingredients modulating changes in the composition and activities of the gut microbiome [82]. Prebiotics are often considered to be a subset of dietary fiber; however, not all dietary fiber has prebiotic qualities [36]. Prebiotic fiber is naturally found in foods such as garlic, asparagus, leek, chicory root and onions [26]. Benefits of prebiotics include: (i) stimulating bacterial production of short chain fatty acids; (ii) a selective modification of gut microbiota composition, reduction of the luminal pH, inhibition of pathogen growth; (iii) improvement in glucose and lipid metabolisms [26]. We lack well-designed studies to evaluate the effects of prebiotic fiber on NAFLD. A review article reported that in patients with T2D and hyperlipidemia who consumed either inulin or fructooligosaccharide (FOS) supplements (prebiotic fiber), improvements in the TG (6–20% reduction), cholesterol (14–27% reduction) levels, or both were noted [83]. Oligofructose supplementation for three months significantly reduced body weight in overweight and obese adults, decreased self-reported calorie intake and improved glycemia [84]. Only one pilot study has assessed the effect of prebiotic fiber in NAFLD. In this study, seven NASH patients received 16 g/day of oligofructose in a randomized, double-blinded, crossover study. The results showed a significant decrease in serum ALT and aspartate aminotransferase (AST) levels, and an improvement in insulin level after four weeks [85].

### 5.4. Omega-3 Interventions

Several clinical studies have evaluated the impact of algal or marine omega-3 PUFAs on hepatic endpoints in NAFLD [47]. A recent study documented that marine omega-3 PUFA modulate hepatic lipid composition and TAG metabolism, and elevate mediators with anti-inflammatory features. All these modulations are beneficial in NAFLD treatment [86,87]. One of the first studies investigating the impact of omega-3 PUFA dates back to 2006 [88]. In this study, Cappani et al. demonstrated that fish oil (1 g/day for 12 months) as compared to control, resulted in a 60% improvement in risk factors such as a significantly decreasing the serum AST), ALT, γ-glutamyl transpeptidase (GGT), improving liver steatosis and reducing the *n-*6/*n-*3 ratio (*p* = 0.0001) [88]. Since that time, a series of clinical trials and pilot studies have also supported the beneficial effects of omega-3 PUFAs with results on liver enzymes and hepatic steatosis. In 2008, two studies investigated the effect of calorie restricted diets with and without fish oil consumption [89,90]. Findings showed that fish oil in addition to calorie restriction was more protective than calorie restriction alone for the treatment of NAFLD. In another study, omega-3 PUFAs (2.7 g/day for 12 months) reduced plasma TAG level by 4.3% compared with baseline data, but had no effect on hepatic steatosis, inflammation, ballooning, or fibrosis score, and showed no improvement in liver enzymes levels, insulin resistance or proinflammatory markers [91]. Recently, two RCTs [92,93] used flaxseed oil as a source of omega-3 PUFAs in patients with NAFLD. One study showed a significantly greater reduction in liver enzymes, hepatic steatosis and fibrosis scores in flaxseed plus lifestyle modification compared to lifestyle modification alone [93]. In another study, Noguiera et al. used a mix of flaxseed oil and fish oil (0.945 g *n-*3/day) and compared the results with mineral oil (2 mL/day). No histological improvement was found after a six-month intervention, except an improvement in lobular inflammation in the group receiving mineral oil [92]. Among all published reports using omega-3 PUFAs for NAFLD treatment, only five studies suggested that omega-3 PUFAs improved NAFLD independent of weight loss. Taken together, the data are discordant and larger well-designed clinical trials for longer periods are still needed to fully evaluate the beneficial effects of omega-3 PUFAs in NAFLD.

### 5.5. Low-Fat vs. Low-CHO Interventions

Low-CHO diets have become a common strategy for weight management across a variety of conditions. Although, a few studies have demonstrated that low CHO diets provide initial benefits on insulin resistance and liver fat, these results have not been sustainable in the long-term [94,95]. In 2011, Haufe et al. [96] performed a six-month trial comparing the effects of reduced fat and reduced CHO hypocaloric diets on intrahepatic fat assessed by magnetic resonance tomography in overweight and obese individuals. The results showed that a prolonged intervention with either low-CHO or low-fat diets had same effect on intrahepatic lipid accumulation, independent of visceral fat loss and changes in insulin sensitivity [96]. Another three-month intervention study was conducted in obese subjects with two intervention groups—one group received a calorie-restricted diet (1100 kcal/day) with low-CHO content (<50 g/day) and the second group followed an isocaloric high-CHO diet (>180 g/day). The result after 48 h showed an almost 30% reduction in intrahepatic TG in patients on the low-CHO diet and only a 10% decrease on the high-CHO diet. The decrease of intrahepatic TG at the end of the study was similar in the two groups (40–50% reduction) [95]. In five biopsy-proven NAFLD patients, a six-month low-CHO ketogenic diet (CHO less than 20 g per day) resulted in a significant improvement in steatosis and inflammatory response (*p* = 0.02) with a trend toward improvement in fibrosis [97]. Although interesting, these results are inconclusive given the absence of a comparative arm and the possibility that improvement may have been due to weight loss alone and not the particular diet. In another study, De Luis et al. [98] explored the effects of either a low-CHO or low-fat hypocaloric diet in NAFLD and obese patients for three months. In the NAFLD group, the low-fat diet showed weight loss had an improvement in anthropometric measurements (BMI, weight, fat mass), cardiovascular risk factors (blood pressure, TG, LDL- and total cholesterol levels) and liver enzymes (ALT, AST, GGT). The low-CHO diet resulted in an improvement in same parameters without statistical changes in AST [98]. A recent meta-analysis study supports that both low/moderate fat (≤30% of daily calorie intake) and moderate CHO (≤45% of daily calorie intake) diets have a similar beneficial effect on liver function by reducing ALT and AST levels [99].

Thus far, the limited available evidence supports the absence of a difference between low-CHO and low-fat diets as long as they are both calorie-restricted diets [96]. Long-term, interventional studies comparing these two diets, low-CHO and low-fat diets, are needed with an assessment of hepatic outcomes in patients with NAFLD.

### 5.6. Probiotics

Altered gut microbiota is associated with obesity, a risk factor for NAFLD. In animal studies, probiotics have been shown to prevent liver fibrosis [100]. In humans, Wong et al. demonstrated that patients with biopsy proven NASH treated with probiotics (*Lactobacilli* strains plus *Bifidobacterium bifidum*) compared with usual care had significantly lower intrahepatic triglyceride content measured by proton-magnetic resonance spectroscopy, independent of effect on BMI, waist circumference glucose or lipid levels [101]. Aller and colleagues investigated the impact of probiotic supplementation in 28 adults with NAFLD over three months. The probiotic intervention was comprised of 500 million colonies of *Lactobacillus bulgaricus* and *Streptococcus thermophiles*. Therapy was associated with a significant reduction in liver enzymes (ALT, AST and GGT; *p* < 0.05) compared to the placebo group. No significant changes were found in anthropometrics and cardiovascular risk factors between groups [102]. Only one study evaluated an intervention using a probiotic food in NAFLD [103]. This randomized, double-blind, placebo-controlled clinical trial showed that consuming a probiotic yogurt with *L. acidophilus* La5 and B. lactis Bb12 for eight weeks in NAFLD patients reduced hepatic aminotransferases, and total and LDL cholesterol compared to patients consuming conventional yogurt [103]. In summary, the limited available data support the benefit of some probiotics, presumably related to modulation of liver function and reduction in dysbiosis, hepatic fat and TNF-α production. The translation of these findings to clinical practice is limited by the small number of studies and the variations in the probiotic strains, doses, and duration of the intervention period chosen across studies.

## 6. Our Recommendations

Weight loss achieved through lifestyle modification is the mainstay of therapy for NAFLD. Unfortunately, the sustainability of weight loss is poor and recidivism is frequently encountered, resulting in inadequate disease control. In this review we aimed to highlight the potential therapeutic role of a “high quality healthy diet” to improve liver steatosis and metabolic dysfunctions in patients with NAFLD above and beyond the known benefit of caloric restriction and weight loss. Our suggested dietary recommendations to manage NAFLD using dietary interventions include a eucaloric diet that is focused in the following principles:

(a) **Moderate to high CHO intake:** The recommended CHO intake is 45–65% of total daily calories, with a low preference for simple and unrefined sugar such as fructose-containing beverages and food; High preference for whole grains and low glycemic index foods. This improves hepatic fat accumulation and decreases hepatic fat accumulation.
(b) **Low to moderate fat intake:** The recommended fat intake is below 30–35% of total calories with a low preference for saturated and trans fat intake; High preference for healthy fat intake (MUFA and omega-3 PUFAs) found for example in olives, olive oil, seeds, nuts and fatty fish. This promotes glycemic control and may reduce inflammatory responses.
(c) **Protein intake:** The recommended protein intake is 15–20% of total daily calories, with minimization of red meat intake particularly processed meat, and increased consumption of poultry, fish, low- or non-fat dairy products and a blend of vegetable protein sources (beans and legumes). This improves cardiovascular risk factors and insulin sensitivity and decreases the risk of morbidity and mortality.
(d) **Fiber and antioxidant intake:** Increased consumption of fruits and vegetables, with more focus on prebiotic fiber. This provides beneficial effects on hepatic metabolism, which may be mediated through gut microbiome changes and reduction in calorie intake.
(e) Consider daily intake of probiotic enriched yogurt, containing *L. acidophilus*.

At this time, we do not have a definitive answer for the optimal proportions of total macronutrients (CHO, fat and protein) in the diet of NAFLD patients. A dietary composition based on the above recommendations may be of benefit in NAFLD, and overlaps with the Mediterranean Diet, which the EASL–EASD–EASO Clinical Guidelines have also recommended as the diet of choice for NAFLD treatment. Future studies evaluating the therapeutic effects of sustainable isocaloric dietary compositional changes will further increase our knowledge in this area.

## Figures and Tables

**Table 1:** Food group distribution in DASH and Mediterranean diets.

DASH Diet		Mediterranean Diet	
Food Group	Daily Servings	Food Group	Daily Servings
Whole grains	7–8	Whole grains, vegetables, fruits, seeds, olive oil, beans, nuts, legumes	Base every meal on these foods
Vegetables	4–5		
Fruits	4–5		
Dairy, low-fat or non-fat	2–3		
Lean meats, poultry, fish	2 or fewer	Fish, seafood	Eat at least twice a week
Nuts, seeds, dry beans	4–5 per week	Poultry, eggs, yogurt, cheese	Eat moderate portions daily to weekly
Fats and oils	2–3	Red meats	<2 servings/week
		Processed Meats	<2 servings/week
Sweets	5 per week	Sweets	<1 serving/week

## Appendix A

**Table A1:** Published clinical trials on the effect of dietary compositions and patterns in adult patients with NAFLD.

*Mediterranean Diet*							
Author/Year	Method of Diagnosis	Study Design, No. of Cases	Intervention	Hepatic Fat Outcomes, Imaging Modality	Results	Compliance and Adherence	Drop-Out Rates
Ryan et al. 2013 [64]	biopsy	Randomized, cross-over trial; 12 obese, non-diabetic NAFLD pts	6 weeks, MD vs. standard low fat/high CHO (LFHC) Diet	1H-MRS	39% reduction in hepatic steatosis in MD group vs. 7% reduction in LFHC diet; A significant improvement in insulin sensitivity (HOMA-IR and circulating insulin level) and a significant reduction in systolic blood pressure only in MD group independent of weight loss; No difference in serum ALT and GGT; No difference in body weight.	Not reported	None
Trovato et al. 2015 [104]	US	Single arm trial; 90 overweight, non-diabetic NAFLD pts	6 months, MD	US	A significant decrease in liver steatosis and HOMA-IR; No significant change in ALT (baseline values were in normal range); the effect of adherence to MD was significantly observed at the sixth month; BMI significantly reduced	Not reported	None
Abenavoli et al. 2015 [105]	US	Randomized controlled trial; 30 overweight NAFLD pts	6 months, Group A: a personalized MD Group B: a personalized MD + Realsil complex * daily Group C: No treatment	NE	MD alone or in association with Realsil complex showed a significant improvement in BMI, waist circumferences, total cholesterol and TG; A significant reduction in HOMO-IR in group B pts; Significant decrease in weight.	Not reported	None
***DASH Diet***							
Razavi Zade et al. 2016 [74]	US	Randomized controlled trial; 60 overweight and obese NAFLD pts	8 weeks, calorie-restricted DASH diet vs. control calorie-restricted diet Calorie restricted diet: 52–55% CHO, 16–18% protein and 30% fat	NE	A significant reduction in weight, BMI, ALT, AST, and improvement in insulin sensitivity in DASH diet group compared to control group; Significant reduction in inflammatory response (hs-CRP and MDA), an increase in antioxidant profiles (NO and GSH) in DASH group compared to control.	Not reported	6 out of 60
***Fiber Intervention (Soluble, Prebiotic)***							
Daubioul et al. 2005 [85]	biopsy	Randomized, double blind cross-over trial; 7 NASH pts	8 weeks, 16 g/day oligofructose (OFS) vs. placebo (maltodextrin)	US	A significant decrease in serum ALT, AST and insulin level in OFS group; No significant changes in liver size and liver fatty infiltration; Effects on body weight not reported	Not reported	Not reported
***Omega-3 Interventions***							
Hatziolios et al. 2004 [106]	US	Clinical trial; 72 NAFLD pts with dyslipidemia	24 weeks, Group A: 15 mL/day Fish oil (EPA 2.25 g/day + DHA 1.58) Group B: orlistat intervention Group C: atorvastatin intervention	US, biopsy	35% improvement in resolution of hepatic steatosis in group A, 61% in group B, and 86% in group C; A significant reduction in liver transaminases in all groups; No significant change in BMI	Not reported	1 out of 73
Capanni et al. 2006 [88]	US	Pilot trial; 56 NAFLD pts	12 months, 1g/day *n*-3 PUFAs (EPA and DHA in the ratio of 0.9/1.5 respectively)	US	Serum ALT, AST, GGT, TG and fasting glucose significantly decreased in intervention group; A significant reduction in *n-*6/*n-*3 PUFA ratio and improvement in hepatic steatosis in treated group; No change in BMI.	Not reported	None
Tanaka et al. 2008 [107]	biopsy	Single arm pilot trial; 23 NASH pts	6 months, 2.7 g/day EPA	biopsy (only in 7 pts)	A significant improvement serum ALT, FFAs; a significant reduction in hepatic oxidative stress factors; An improvement in liver histology in 6 pts; No change in weight, glucose, insulin and adiponectin levels; No significant difference in body weight.	Not reported	None
Vega et al. 2008 [108]	MRS	Sequential design;	4 weeks of placebo followed by 8 weeks treatment with 9 g/day fish oils	MRS	46% reduction in plasma TG, 21% reduction in LDL and IDL and 15% reduction in total apo-B; No significant changes in intrahepatic TG by fish oil treatment; No significant change in body weight	Not reported	5 out of 22
Zhu et al. 2008 [90]	US	Randomized, controlled trial; 134 NAFLD pts with hyperlipidemia	24 weeks, 6 g/day *n-*3 PUFA from seal oils vs. placebo group; both group received AHA-recommended diet AHA-recommended diet: 50% CHO, 20% protein and 30% fat, hypocaloric diet (25–30 kcal/kg BW) was advised to overweight pts	US	A significant reduction in ALT, TG and LDL-cholesterol in PUFA group and a tendency toward improvement in GGT, AST, HDL- and total cholesterol in the both groups; A normalization in ultrasonographic evidence; No significant changes in BW and fasting glucose	Not reported	10 out of 134
Spadaro et al. 2008 [89]	US	Randomized, controlled trial; 40 pts with hyperlipidemia	6 months, 2 g/day *n*-3 PUFAs + AHA-recommended diet vs. AHA-recommended diet alone	US	An improvement in ALT, TG and TNF-α in PUFA group; A normalization in ultrasonographic features of NAFLD; BMI levels were decreased in both groups.	Not reported	4 out of 40
Scorletti et al. 2014 [109] WELCOME study	Radiological- or biopsy-proven	Randomized double blind placebo controlled trial; 95 NAFLD pts	18 months, 4 g/day *n-*3 PUFA (DHA + EPA) vs. placebo (4 g/day olive oil)	MRS	A marginal reduction in intrahepatic TG in PUFA group; Change in body weight not reported.	Not reported	8 out of 103
Sanyal et al. 2014 [91]	biopsy (NAS > 40)	Randomized, controlled trial; 234 NAFH pts	12 months, 1.8 g/day EPA-E (low-dose) vs. 2.7 g/day EPA-E (high-dose) vs. placebo	biopsy	35–40% reduction in NAS, no fibrosis worsening, without any difference between groups; A significant reduction in TG levels, but no effects of EPA-E on biochemical and histological features; No significant change in body weight	Overall compliance rate around 90%	15 out of 243
Argo et al. 2015 [102]	biopsy	Randomized, controlled trial; 34 non-cirrhotic NASH pts	12 months, 3 g/day *n*-3 PUFAs vs. placebo	biopsy, MRI	*N-*3 PUFAs group showed a greater reduction in intrahepatic TG in individuals with increased or stable BW; No improvement in NAS score (≥2 without fibrosis progression); No effect on hepatocellular injury and IR; No significant change in body weight.	Not reported	7 out of 41
Dasarathy et al. 2015 [110]	biopsy	Randomized, double blinded, placebo-controlled trial; 37 well-controlled diabetic NASH pts	48 weeks, 2.16 g/day EPA + 1.44 g/day DHA vs. placebo (corn oil)	biopsy	No change in liver enzymes, BW and body composition; In placebo group, hepatic steatosis and NAS score improved and lobular inflammation worsened but no changes in PUFA group; An increase in HOMA-IR in PUFA group	Not reported	None
Li et al. 2015 [111]	biopsy	Prospective, randomized, controlled, unblended trial; 78 NASH pts	6 months, 50 mL/day *n*-3 PUFA (1:1 ratio EPA:DHA) vs. placebo (prescribed normal saline) Recommended diet for both group: low-fat/low-CHO diet + 30 min modest physical exercise at least 5 days/week	biopsy	A significant reduction in liver function (ALT, AST), serum TG, total cholesterol and metabolic profiles in PUSA group; A reduction in BMI in both groups; A significant improvement in severity of NASH in treated group	Not reported	None
Nogueira et al. 2016 [92]	biopsy	Randomized, double blinded, controlled trial; 50 NASH pts	6 months, 0.945 g/day *n*-3 PUFA (64% ALA + 16% EPA + 21% DHA) vs. placebo (mineral oil)	biopsy	A significant increase in plasma *n-*3 PUFA in both group; NAS score improvement/stabilization was significantly correlated with increased plasma *n-*3 PUFAs in both group; A significant reduction in TG level in PUFA group; BMI did not change significantly	Not reported	10 out of 60
***Low-Fat Diet*** **vs. *Low-CHO Diet***							
Petersen et al. 2005 [112]	1H-MRS	Clinical trial; 8 obese, diabetic pts with non-alcoholic steatosis	12 weeks moderately hypocaloric very-low-fat (3%) followed by 4 weeks of isocaloric diet Group 1: patients (*n* = 8) Group 2: healthy controls (*n* = 10)	1H-MRS	A 8 kg weight loss significantly improved glucose metabolism; A 81% reduction in intrahepatic TG	Not reported	None
Tendler et al. 2007 [97]	biopsy	Single arm pilot study; 5 obese NAFLD pts	3 months, low-CHO ketogenic diet (<20 g/day CHO) with nutritional supplementation (free of any weight loss-induced formula) No restriction on the amount of calorie intake	biopsy	A 12.8 kg weight loss after treatment; A significant improvement in liver histology in 4 pts	Not reported	None
Kirk et al. 2009 [95]	1H-MRS	Randomized clinical trial; 22 obese pts (NAFLD pts: 50% of pts in high CHO diet, 58% of pts in low CHO diet)	11 weeks, Group 1: high CHO diet (>180 g/day) Group 2: low CHO diet (<50 g/day)	1H-MRS	After 48 h, an almost 30% reduction in intrahepatic TG on low-CHO diet and only 10% decrease on high-CHO diet; A decrease of intrahepatic TG at the end of the study in both groups (40–50% reduction); 7% weight loss in both groups	Subjects in both groups were compliant with the diet	None
De Luis et al. 2010 [98]	Elevated liver enzyme (ALT)	Randomized clinical trial; 28 obese NAFLD pts	3 months, Group 1: Pts with normal ALT activity (control; *n* = 134) Group 2: NAFLD pts (*n* = 28) Diet I: low fat Diet II: low CHO	NE	In NAFLD group, low-fat diet showed weight loss and an improvement in anthropometric measurements, cardiovascular risk factors and liver enzymes; and low-CHO diet resulted in an improvement in same parameters without statistical changes in AST	Not reported	None

**Abbreviations:** NAFLD, non-alcoholic fatty liver disease; pts patient; MD, Mediterranean diet; H-MRS, H-Magnetic Resonance Spectroscopy; US, Ultrasound; HOMA-IR, Homeostatic Model Assessment of Insulin Resistance; ALT, alanine aminotransferase; NE, not evaluated; TG, triglycerides; CHO, carbohydrate, GGT, γ-glutamyl transpeptidase; hs-CRP, high-sensitivity C-reactive protein; MDA, malondialdehyde; NO, nitric oxide; GSH, glutathione; NASH, non-alcoholic steatohepatitis; EPA, eicosapentaenoic acid; DHA, docosahexaenoic acid; BW, body weight; AHA, American Heart Association; TNF, tumor necrosis factor; NAS, NAFLD activity score; MRI, magnetic resonance imaging; IR, insulin resistance; ALA, α-Linolenic acid; IDL, intermediate density lipoprotein; apo-B, apolipoprotein B; BMI, body mass index; AST, aspartate aminotransferase. * Realsil complex: a complex of silybin, phosphatidylcholine and vitamin E.
